# The Curability of Uterine Displacements

**Published:** 1881-12

**Authors:** 


### THE CURABILITY OF UTERINE DISPLACEMENTS.

Dr. Paul F. Munde, of New York, finding that the text-books either entirely omit all mention of the possibility of permanently curing displacements of the uterus by any of the methods in use, or give but vague statements on the subject, and impressed with the importance of having some positive conclusions on this matter, both for the sake of the patient and the satisfaction of the physician, in a paper presented to the London Congress has analyzed the numerous cases of displacements which have come under his care (895), and arrives at the following conclusions :—

1. Displacements of the uterus are permanently curable in the large majority of cases only when recent, or when a complete tissue metamorphosis, as occurs during pregnancy and after parturition, takes place.

2. Chronic cases (of more than a year's standing) are but rarely curable permanently, except occasionally under the last-named circumstances. Apparent cures reported by some authors and witnessed by many physicians, soon show themselves to have been but temporary.

3. Pess ıries form unquestionably the most practical, rational, and (temporarily) the most efficient means of treating uterine displacements. Cures are but rarely accomplished by them.

4. Medicated, chiefly astringent, tampons, intelligently applied every day by the physician, give the best chances for permanent cure. This is particularly true of prolapsus, but holds good for all forms.

5. Electricity locally applied deserves more extended application.

6. All methods should be persevered in for months and years before success is to be expected.—*Med. News and Abstract.*

